# Hcmv-miR-UL148D regulates the staurosporine-induced apoptosis by targeting the Endoplasmic Reticulum to Nucleus signaling 1(ERN1)

**DOI:** 10.1371/journal.pone.0275072

**Published:** 2022-09-26

**Authors:** Abhishek Pandeya, Raj Kumar Khalko, Sukhveer Singh, Manish Kumar, Sunil Babu Gosipatala

**Affiliations:** 1 Department of Biotechnology, Babasaheb Bhimrao Ambedkar University, Lucknow, Uttar Pradesh, India; 2 Developmental Toxicology Laboratory, CSIR-Indian Institute of Toxicology Research (CSIR-IITR), Lucknow, Uttar Pradesh, India; 3 National Heart Lung and Blood Institute, National Institute of Health, Bethesda, Maryland, United States of America; Shanxi University, CHINA

## Abstract

The propensity of viruses to co-opt host cellular machinery by reprogramming the host’s RNA-interference machinery has been a major focus of research, however, regulation of host defense mechanisms by virus-encoded miRNA, is an additional regulatory realm gaining momentum in the arena of host-viral interactions. The Human Cytomegalovirus (HCMV) miRNAs, regulate many cellular pathways alone or in concordance with HCMV proteins, thereby paving a conducive environment for successful infection in the human host. We show that HCMV miRNA, hcmv-miR-UL148D inhibits staurosporine-induced apoptosis in HEK293T cells. We establish that ERN1 mRNA is a bonafide target of hcmv-miR-UL148D and its encoded protein IRE1α is translationally repressed by the overexpression of hcmv-miR-UL148D resulting in the attenuation of apoptosis. Unlike the host microRNA seed sequence (6–8 nucleotides), hcmv-miR-UL148D has long complementarity to 3’ UTR of ERN1 mRNA resulting in mRNA degradation. The repression of IRE1α by the hcmv-miR-UL148D further downregulates Xbp1 splicing and c-Jun N-terminal kinase phosphorylation thus regulating ER-stress and ER-stress induced apoptotic pathways. Strikingly, depletion of ERN1 attenuates staurosporine-induced apoptosis which further suggests that hcmv-miR-UL148D functions through regulation of its target ERN1. These results uncover a role for hcmv-miR-UL148D and its target ERN1 in regulating ER stress-induced apoptosis.

## Introduction

Human cytomegalovirus (HCMV) belongs to the β-herpesvirus family and infects most organs and tissues. HCMV is transmitted in almost every part of the world and more than 90% of the general population is an HCMV carrier [1, 2]. HCMV infection triggers numerous diseases like inflammation, atherosclerosis, Crohn’s disease, pneumonia, and various cancers [3–7] by disrupting the normal physiological activity of host cells, particularly apoptosis, autophagy, and immune response [8, 9]. Moreover, HCMV infection can trigger life-threatening diseases in immunosuppressed individuals [10]. Many viruses modulate the cellular defense mechanisms by targeting and/or regulating their host proteins, through different molecules such as proteins, RNAs and miRNAs thereby establishing successful infection in their hosts. Like many viruses, human cytomegalovirus (HCMV) encodes miRNAs, reported to play a regulatory role in various cellular pathways, including apoptosis [11–19]. The HCMV genome is around 230 kb and encodes more than 26 mature microRNAs (miRNAs) [8]. Post-transcriptional gene regulation by miRNAs is achieved through specific base‑pairing between nucleotides located at positions 2–7 at the 5’ end of the miRNA (seed region) and the 3’ untranslated region (3’UTR) of target mRNAs, leading to the translational repression or decay of target mRNA [20].

Apoptosis is an innate immune mechanism, causing the death of viral infected cells, preventing viral replication and propagation, which is broadly classified into extrinsic, intrinsic, mitochondrial-dependent and endoplasmic reticulum (ER) stress-induced pathways depending on the initiator molecules of this pathway [21, 22]. The high viral load results in perturbations in the ER, and to resolve this stress, ER initiates signaling pathways collectively termed as unfolded protein responses (UPR). HCMV modulates all the three branches of UPR signaling viz., PKR-like ER kinase (PERK), activating transcription factor-6 (ATF-6), and inositol requiring enzyme- 1(IRE1) during its infection [23]. At initial periods of HCMV infection, all the three pathways get activated, however, later, they are modulated in such a way that the outcome favors HCMV replication [23]. Though most of the HCMV antiapoptotic proteins act at mitochondrial level [24], or on caspases [25], however, few also act on the UPR [26, 27]. These studies clearly indicated the role of HCMV infection regulating the immune response, though molecular mechanisms of apoptosis have been poorly characterized.

Our previous *in silico* analysis predicted that hcmv-miR-UL148D can target the ERN1 mRNA, suggesting HCMV miRNA role in the ER-stress response [8]. The ERN1 encodes a transmembrane protein kinase, inositol requiring enzyme 1 (IRE1), which switches between XBP1 splicing and ER-stress induced apoptosis depending on the time and extent of the UPR [28]. The hcmv-miR-UL148D was first discovered from the studies of Pfeffer *et al*. in 2005 [29], highly expressed during latent infections [30], and regulates cellular genes. It subvert the immune response by translationally repressing the immune genes, RANTES [33], IL-6 [32] and also acts as anti-apoptotic by targeting IEX-1 [12], moreover it facilitates the HCMV latency [18]. Hcmv-miR-UL36-5p targets adenine nucleotide translocator 3 to inhibit apoptosis [14]. Previous studies show the importance of other HCMV miRNA, miR-US5-1, and miR-UL112-3 in regulation of apoptosis through FOXO3a [19]. However, some HCMV microRNA also promotes apoptosis like miR-US4-1, and miR-US4-5p by targeting the QARS and p21-activated kinase 2 respectively [16, 17]. Further, hcmv-miR-US25-1 also induce apoptosis by regulating the oxidized low density lipoprotein [31]. We recently showed the importance of hcmv-miR-UL70-3p in regulation of apoptosis by targeting MOAP1 [32]. Thus, HCMV microRNA either function as pro-apoptotic or anti-apoptotic which suggests that more investigation is required to elucidate its role by identifying and characterizing more cellular targets.

In this study, we show that hcmv-miR-UL148D inhibits staurosporine-induced apoptosis in HEK293T cells through regulating the expression of ERN1. We also validate that hcmv-miR-UL148D targets 3′UTR of ERN1 and leads to its degradation through RNAi machinery. Further, degradation of ERN1 mRNA results into low protein level of IRE1α which directly regulates H_2_O_2_ induced apoptosis by modulating XBP1 splicing, and phosphorylation status of c-Jun. These findings establish a novel target and mechanism of HCMV encoded miRNA, hcmv-miR-UL148D that has anti-apoptotic function by repressing its cellular target ERN1.

## Materials and methods

### Cells

HEK293T cells (ATCC # CRL-3216) cultured in Dulbecco’s modified eagle’s medium (DMEM) supplemented with 10% fetal bovine serum (FBS) and 1% antibiotic and antimycotic (Cat No:15240062; Gibco-Life Technologies) were incubated in CO_2_ incubator under the controlled temperature of 37°C with 5% CO_2_. The cells were divided into 4 groups, viz., negative control (without treatment); positive control (staurosporine treated); test groups of the cells transfected with 25nM of hcmv-miR-UL148D mimic followed by the 1μM of staurosporine for 24h [35] and the last group transfected with miR-UL148D mimic along with its inhibitor followed by the staurosporine treatment. The cells transfected with hcmv-miR-UL148D alone were also assayed for apoptotic effects and treated as another control group.

### Transfections and co-transfections

The HEK293T cells seeded in 6 well plates were transfected with 25μM hcmv-miR-UL148D mimic (5’-UCG UCC UCC CCU UCU UCA CCG-3’; assay ID: MC11077; Cat No: 4464066, Life Technologies, USA) and/or its sequence specific inhibitor (Assay ID: MH11077; Cat No: 4464084, Life Technologies, USA) per well using Dharmafect 1 (Cat No: T-2001-03; Dharmacon) as per manufacturer’s instructions. The siRNA of ERN1 (sequence 5’ AUC UGU GAU CAA UGA GAA AUC UCA CAC 3’; Design ID: hs.Ri. ERN1.13.2) designed against the 3’UTR of ERN1 at the position of 505–547 nt was procured from the Integrated DNA Technologies, Inc., Coralville, IA, USA. The co-transfections of hcmv-miR-UL148D mimic, miR-UL148D mimic along with its sequence specific inhibitor and pEZX-MT06-3′UTR^WT^-ERN1 and pEZX-MT06-3′UTR^DEL^-ERN1 were done through lipofectamine 3000 (Cat No. L3000008; Invitrogen, USA) as per manufacturer’s instructions.

### Flow cytometry

To investigate the effect of hcmv-miR-UL148D on apoptosis in different experimental groups of cells mentioned above were measured for apoptotic cells by using Annexin-V conjugated with Alexa Fluor 488 conjugate (Cat No: A13201; Invitrogen) and Propidium Iodide (PI) (Cat No: P1304MP; Invitrogen) by a flow cytometer (FACS Canto; BD Biosciences). Briefly, after transfecting the cells with respective hcmv-miR-UL148D, hcmv-miR-UL148D with its sequence specific inhibitor and inducing the apoptosis through staurosporine, the cells were collected and washed with chilled PBS twice. The cells pellet was resuspended in 400μl of 1✕annexin binding buffer (Cat No: V13246; Invitrogen), incubated with 5μl annexin V and 10μl of PI in the dark at room temperature for 15 min, and the apoptotic cells were measured through flow cytometer.

### DAPI analysis

The nucleus morphology was analyzed through 4’,6-diamidino-2-phenylindole (DAPI) staining in the 5 different experimental groups containing 1^st^ group negative control (untreated), 4^th^ and 5^th^ group are the cells transfected with the hcmv-miR-UL148D mimic, miR-UL48D mimic along with its inhibitor and then the cell groups 2nd, 4th and 5th groups of cells further treated with the 1μM staurosporine for 24h. The staining was done as per the method described by Chazotte et al. in 2011 [33]. Briefly, the cells were grown on the sterile coverslip for 24h, then they were transfected/ treated as described above. Post 24h of incubation, the cells were fixed with 3.7% formaldehyde, permeabilized with TritonX-100 (0.2% in PBS), stained with Rhodamine Phalloidin (Cat No: R415; Invitrogen) first then the coverslips were mounted with the DAPI mounting media (Cat No: 00-4959-52; Invitrogen). The nuclear morphology showing the chromatin condensation/nuclear fragmentation was analyzed using a confocal microscope.

### Caspase 3/7 assay

The effector caspases, Caspase 3/7 activity was measured in the said experimental groups of cells by Caspase Glo 3/7 assay (Cat No: G8090; Promega) as per the manufacturer’s instructions. Briefly, the cells were seeded at 0.01✕10^6^ cells/well in 96 well white plates and after 24h of incubation, the cells were divided into 5 different groups in which 1^st^ group is negative control (untreated and un-transfected), 3^rd^ and 4^th^ group- transfected with hcmv-miR-UL148D mimic; 5^th^ group is transfected with hcmv-miR-UL148D mimic along with its inhibitor. After 24 h incubation, 2^nd^, 4^th^ and 5^th^ groups of cells were treated with 1μM of staurosporine for 24 h. The Caspase 3/7 activities were detected in these groups of cells by using Caspase Glo 3/7 assay through a luminometer.

### qRT-PCR

Total RNA was isolated from the different experimental groups using the pure link RNA mini kit (Cat No: 12183018A; Invitrogen) as per the manufacturer’s instructions. Reverse transcriptions were performed from 1μg of total RNA using ProtoScript® II first strand cDNA synthesis kit (Cat No: E6560S; New England Biolabs Inc, USA) as per the manufacturer’s instructions. qRT-PCR was performed by using PowerUp™ SYBR™ Green master mix (Cat No: A25742; Applied Biosystems, USA) with the following temperature cycle; UDG activation at 50°C for 2 min, activation of DNA polymerase at 95°C for 2 min, followed by denaturation at 95°C for 15 sec, and annealing/ extension at 60°C for 1 min for 40 cycles. The mRNA expressions in both the control (untreated) and test groups (treated with staurosporine; transfected with miR-UL148D mimic followed by the staurosporine treatment and the cells transfected with the hcmv-miR-UL148D mimic along with its inhibitor followed by the staurosporine treatment) were analyzed using the sets of primers enlisted in the **Table 1** (Integrated DNA Technologies, USA). The relative mRNA expressions were normalized with GAPDH in the corresponding samples, and the results were represented as 2^-ΔΔCt^. All the experiments were performed in triplicates and the results are presented as means ± SEM.

**Table 1:** List of qRT-primers.

Primers	Sequences (5’→ 3’)	References
MOAP1	**F**-**5’-**CACGAGCACTAGATCACGGCTGCTGGA-**3’**	[34]
	**R-5’-**CTGCCACACAGCAGCTCTGGGAGATGCC-**3’**	
ERN1	**F-5’-**CGGGAGAACATCACTGTCCC-**3’**	[35]
	**R-5’-**CCCGGTAGTGGTGCTTCTTA-**3’**	
BAK1	F-**5’-**GCTCCCAACCCATTCACTAC-**3’**	[36]
	**R-5’-**TCCCTACTCCTTTTCCCTGA-**3’**	
Caspase 3	**F-5’**-TGGATTATCCTGAGATGGGTTT-**3’**	[37]
	**R-5’-**TTGCTGCATCGACATCTGTA-**3’**	
Caspase 7	**F-5’-**GTAACCCGTTGAACCCCATT-**3’**	
	**R-5’-** CCATCCAATCGGTAGTAGCG**-3’**	
Caspase 9	**F-5’-**TGCTGAGCAGCGAGCTGTT-**3’**	[38]
	**R**-**5’-**AGCCTGCCCGCTGGAT-**3’**	
P53	**F-5’-**CCACCATCCACTACAACTACAT-**3’**	
	**R-5’-** CAAACACGGACAGGACCC-**3’**	
GAPDH	**F-5’-**ACATCGCTCAGACACCATG-**3’**	[39]
	**R-5’-**TGTAGTTGAGGTCAATGAAGGG-**3’**	
XBP1u	**F-5’-**CAGACTACGTGCACCTCTGC-**3’**	[40]
	**R-5**’-CTGGGTCCAAGTTGTCCAGAAT-3’	
XBP1s	**F-5’**- GCTGAGTCCGCAGCAGGT-**3’**	
	**R-5’-**CTGGGTCCAAGTTGTCCAGAAT-**3’**	

Further, the expression of hcmv-miR-UL148D mimic in the transfected cells was detected by the real-time PCR. The cDNA was prepared from the 1μg of total RNA isolated from the transfected and treated cells by using the stem-loop primers as 5’- CTCAACTGGTGTCGTGGAGTCGGCAATTCAGTTGAGCGGAGAAG-3’ designed as per Kramer et al., 2011 [41] specific for the miR-UL148D and 5s rRNA. RT-PCR was performed using small RNA specific primers of hcmv-miR-UL148D as Forward 5’- TCGTCCTCCCCTTCTTCA-3’ and reverse 5’-CTCAACTGGTGTCGTGGA-3’ and for 5s rRNA forward 5’-GTCTACGGCCATACCACCTGAAC-3’ and reverse 5’-CTCAACTGGTGTCGTGGA-3’. The relative expression levels of hcmv-miR-UL148D were normalized to those of the 5s rRNA in the corresponding samples and the results are represented as 2^-ΔΔCt^ method.

### Luciferase reporter assays

Luciferase reporter vector constructs of 3’UTR of ERN1, both wild (pEZX-MT06-3’UTR^WT^-ERN1; Cat No: HmiT016794-MT06-03; Genecopoeia) and mutant type (pEZX-MT06-3’UTR^DEL^-ERN1; Cat No: HmiT016794-MT06-04; Genecopoeia) where the binding site (455-480nt) for hcmv-miR-UL148D was deleted, were procured from the Genecopoeia, USA., which contains firefly luciferase as a reporter gene and renilla luciferase as the tracking gene. The HEK293T cells were co-transfected with 1μg of these vectors along with the 25nM of hcmv-miR-UL148D mimic and equimolar concentrations of hcmv-miR-UL148D mimic along with its inhibitor using lipofectamine 3000 (Cat No: L3000008; Invitrogen). After 24h of incubation, both firefly and renilla luciferase activities were measured by using the dual luciferase reporter assay (Cat No. E1910; Promega Inc,). The measurements were done in triplicates and maximum luciferase activity was calculated by normalizing firefly luciferase activity to renilla luciferase activity within each sample, and the results were presented as the mean ± SEM.

### Immunoblotting analysis

The IRE1α protein levels were analyzed by immunoblotting. The total protein was extracted by using RIPA cell lysis buffer, and the protein quantification was done with the bicinchoninic acid (BCA) protein assay kit (Cat No: 443 786–570; G Biosciences, USA). Equal protein concentrations from defined experimental groups were loaded and resolved on the 10% SDS-PAGE, transferred to the polyvinylidene difluoride (PVDF) membrane at 4°C (Merck Millipore Corp). The blotted membrane was incubated with primary monoclonal rabbit antibody for IRE1α (Cat No: 3294S; CST, USA), primary polyclonal rabbit antibody for XBP1 (Cat No: ab198999; abcam); Phospho-JNK1/JNK2 (Thr183, Tyr185) primary monoclonal rabbit antibody (Cat No: 700031; Invitrogen, USA) and for β-actin (Cat No: 4970S; Cell Signaling Technology, USA). Then the blots were further incubated with anti-rabbit secondary antibodies conjugated with horseradish peroxidase (HRP) (Cat No: 7842S; Cell Signaling Technology, USA). The blots were developed using an enhanced chemiluminescence detection system (Cat No: RPN2209; Amersham ECL Western Blotting Detection Reagent), and visualized on Chemiscope (Clinx). The density of each protein band was quantified by using ImageJ software (ver 1.53e).

### Statistical analysis

The statistical analysis was performed with GraphPad Prism 8.0 software (GraphPad Software, San Diego, CA, USA). Data from three independent experiments were recorded as mean ±SEM and used for the statistical analysis. Statistical significance was determined by using ANOVA, and the p values, less than 0.05 were considered as statistically significant.

### Ethical and biosafety approvals

This study was approved by Institutional Ethics Committee (IEC No: 19/BBAU-IEC/2016); Institutional Biosafety Committee (IBSC No: 01/IBSC/BBAU/2016), Babasaheb Bhimrao Ambedkar University, Lucknow—226025 Uttar Pradesh (India).

## Results

### 1. Staurosporine treatment induces the apoptotic marker genes in HEK293T cells

**Fig 1:**
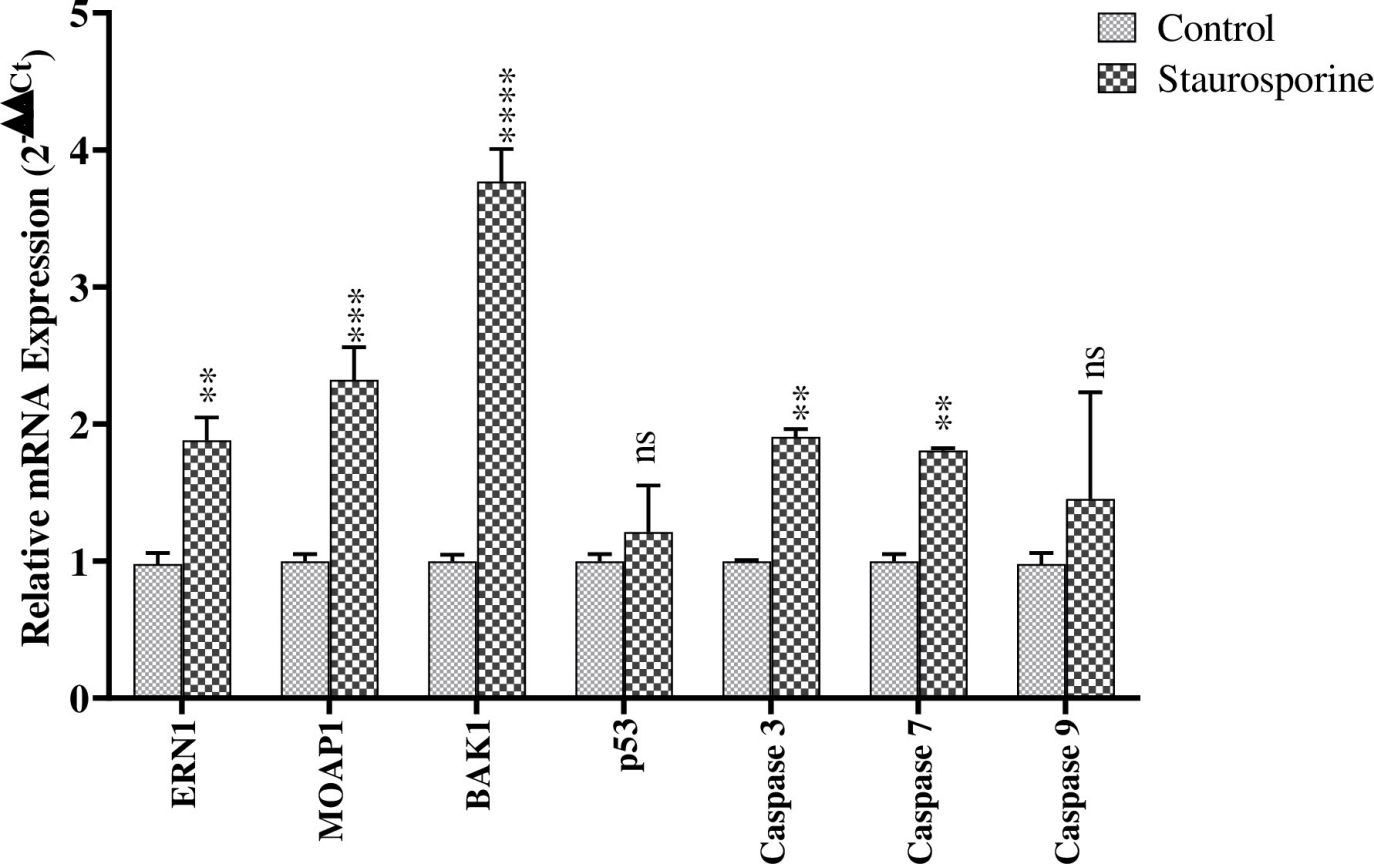
Expression analysis of apoptotic markers in staurosporine treated HEK293T cells: The relative expression of ERN1, MOAP1, BAK1, p53 Caspase 3, Caspase 7 and Caspase 9 mRNAs were analyzed through quantitative reverse transcription polymerase chain reaction. RNA was isolated from staurosporine-treated HEK293T cells and expression of aforesaid genes were quantified and compared with respect to untreated cells. GAPDH was used as a housekeeping control and data plotted as a measure of relative expression. Results represent the mean ± SEM (n = 3 independent experiments) **p<0.01; ***p<0.001; ****p<0.0001; ns = non-significant.

Staurosporine has long been a well-established broad-spectrum inhibitor of protein kinases and is widely used to study intracellular stress-induced apoptosis [42]. Therefore, we examined the apoptosis-inducing potential of staurosporine treatment of HEK293T cells for 24h. We first measured the expression of ERN1 and some other apoptotic gene levels in HEK293T cells after staurosporine treatment [43]. The gene expression analysis through RT-qPCR (list of primers are provided in Table 1) shows that staurosporine treatment significantly induces the mRNA levels of ERN1, MOAP1, BAK1, Caspase-3, and Caspase 7 when compared to the untreated control group, whereas to our surprise p53 and Caspase-9 levels were slightly increased in treated group,however, relative expression compared to control are statistically insignificant **(Fig 1)**. We reasoned that staurosporine-induced apoptosis is cell line dependent and activated signaling pathways [44, 45]. These results suggest that staurosporine-mediated apoptosis in HEK293T cells largely depends upon ERN1, MOAP1, and BAK1, which may be the key initiator molecules for the apoptosis pathway in our model.

### 2. hcmv-miR-UL148D inhibits staurosporine-induced apoptosis in HEK293T cells

To examine the role of hcmv-miR-UL148D, we focused on our previously published *in silico* predictions which suggests that hcmv-miR-UL148D targets the endoplasmic reticulum stress signaling molecule ERN1 [8], which indicates that hcmv-miR-UL148D may have role in apoptosis. To test this hypothesis, we transfected HEK293T cells with hcmv-miR-UL148D mimic and/or inhibitor followed by staurosporine treatment, and apoptosis was assessed through nuclear condensation by DAPI -Rhodamine Phalloidin counterstaining, flow cytometry and Caspase 3/7 measurement.

**Fig 2:**
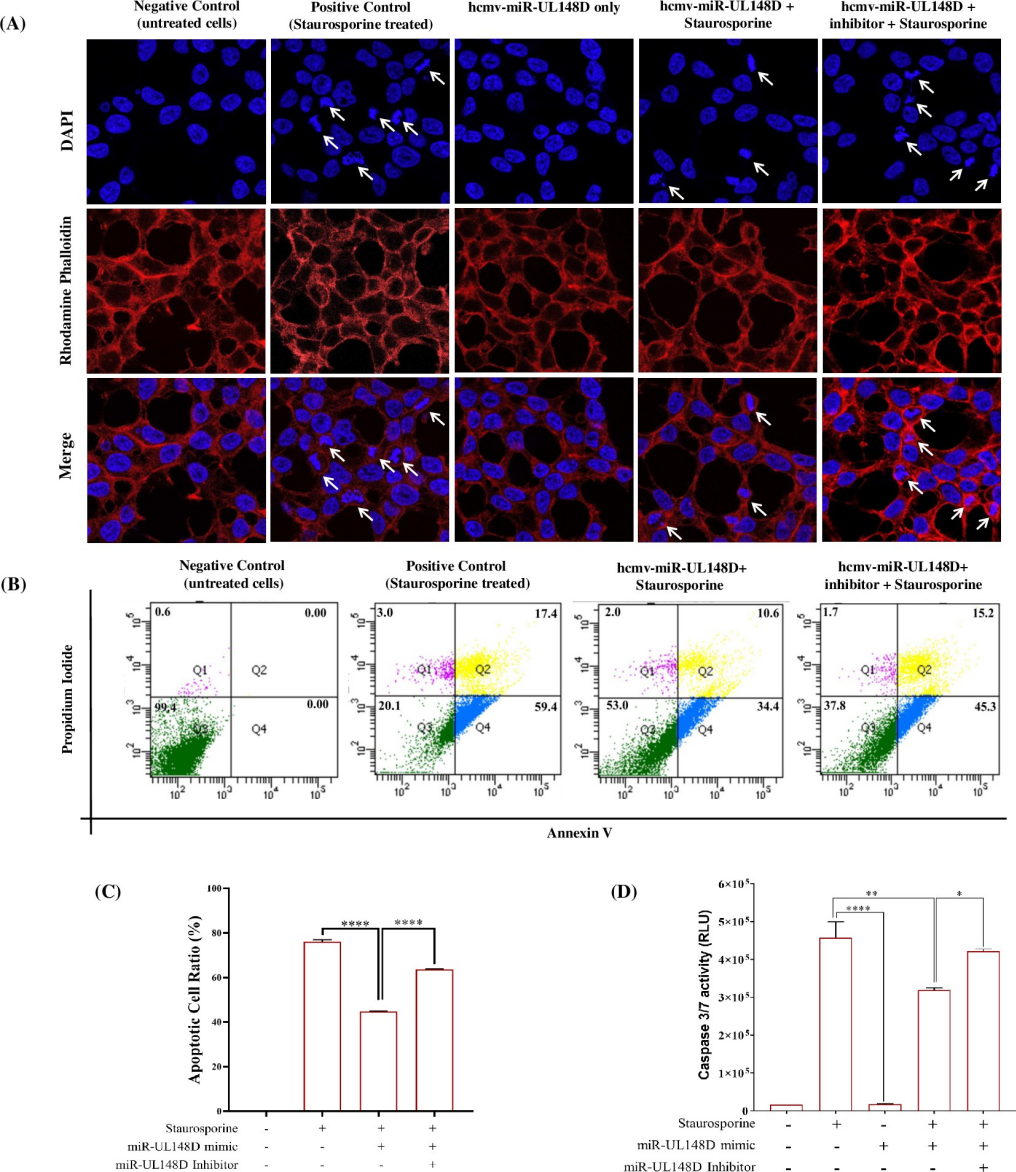
Antiapoptotic effects of hcmv-miR-UL148D in staurosporine-treated HEK293T cells: **(A)** hcmv-miR-UL148D mimic or inhibitor transfected HEK293T cells were treated with staurosporine followed by counterstaining with the DAPI and rhodamine phalloidin. The arrow indicates the significant changes associated with the apoptosis as decrease in the chromatin condensation and nuclear fragmentation by hcmv-miR-UL148D (image acquired at 63X). **(B)** From the same set of groups as in Fig A, cells were stained with propidium iodide and annexin V followed by flow cytometry analysis. The flow cytometry data represented in dot plots show decrement of apoptotic cells by hcmv-miR-UL148D. **(C)** The apoptotic cell ratio was calculated from the flow cytometric data using ImageJ (mean±SEM; ****, p<0.0001). **(D)** HEK293T cells were transfected with hcmv-miR-UL148D mimic or inhibitor then treated with staurosporine. Caspase 3/7 activity was measured from cell lysate using luminescence and expressed as raw luminescence units (RLU). Results represent the mean ± SEM (n = 3 independent experiments) **p<0.01; ***p<0.001; ****p<0.0001.

- **DAPI and Rhodamine Phalloidin counterstaining:** We used microscopy to characterize antiapoptotic activity of hcmv-miR-UL148D. The characteristic features of apoptosis such as chromatin condensation and nuclear fragmentation were analyzed in the mentioned experimental groups of cells through DAPI and Rhodamine Phalloidin counterstaining. The images were captured at 63X through a confocal microscope, and the results show the hcmv-miR-UL148D decreased the chromatin condensation and nuclear fragmentation when compared to the control groups (**Fig 2A**). Blocking the hcmv-miR-UL148D effect with its inhibitor increases the chromatin condensation/nuclear fragmentation, suggesting that the observed inhibition was due to the hcmv-miR-UL148D.
- **Flow cytometry:** The flow cytometry studies show that hcmv-miR-UL148D treatment significantly decreased the staurosporine-induced apoptosis (**Fig 2B**). The apoptotic cell ratio quantification in the different cell groups shows that ectopic expression of hcmv-miR-UL148D downregulated the apoptotic cell ratio from 76.8% to 45%, which is statistically significant. Further, the abrogation of the hcmv-miR-UL148D effect with its inhibitor increases the apoptotic cell ratio from 45% to 60.5%, suggesting hcmv-miR-Ul148D role in apoptotic inhibition (**Fig 2C;** ****p<0.0001).
- **Caspases 3/7 activity:** In continuation, we further measured the Caspase 3/7 activity in the said group of cells in the presence and absence of hcmv-miR-UL148D through Caspase Glo 3/7 assay. The hcmv-miR-UL148D treatment downregulates the caspase 3/7 activity when compared to the control groups (**Fig 2D**). The hcmv-miR-UL148D mimic treatment reduces the Caspase 3/7 activity (RLU is 3.26×10^5^) as compared to the control (staurosporine group the RLU is 4.56×10^5^), decrement of 1.30×10^5^ RLU was observed. The experiments with the hcmv-miR-UL148D inhibitor confirm the Caspase 3/7 level inhibitions by the miR-UL148D. Further, we checked whether the transfection of hcmv-miR-UL148D alone can induce/inhibit any Caspase 3/7 activity and found that the RLU value is 0.22 × 10^5^, which is less when compared to the RLU obtained through the cell group treated with staurosporine (**Fig 2D)**. These results strengthen the argument that the hcmv-miR-UL148D is directly linked to apoptosis regulation in staurosporine treated HRK293T cells.

### 3. ERN1 is a direct target for hcmv-miR-UL148D

**Fig 3:**
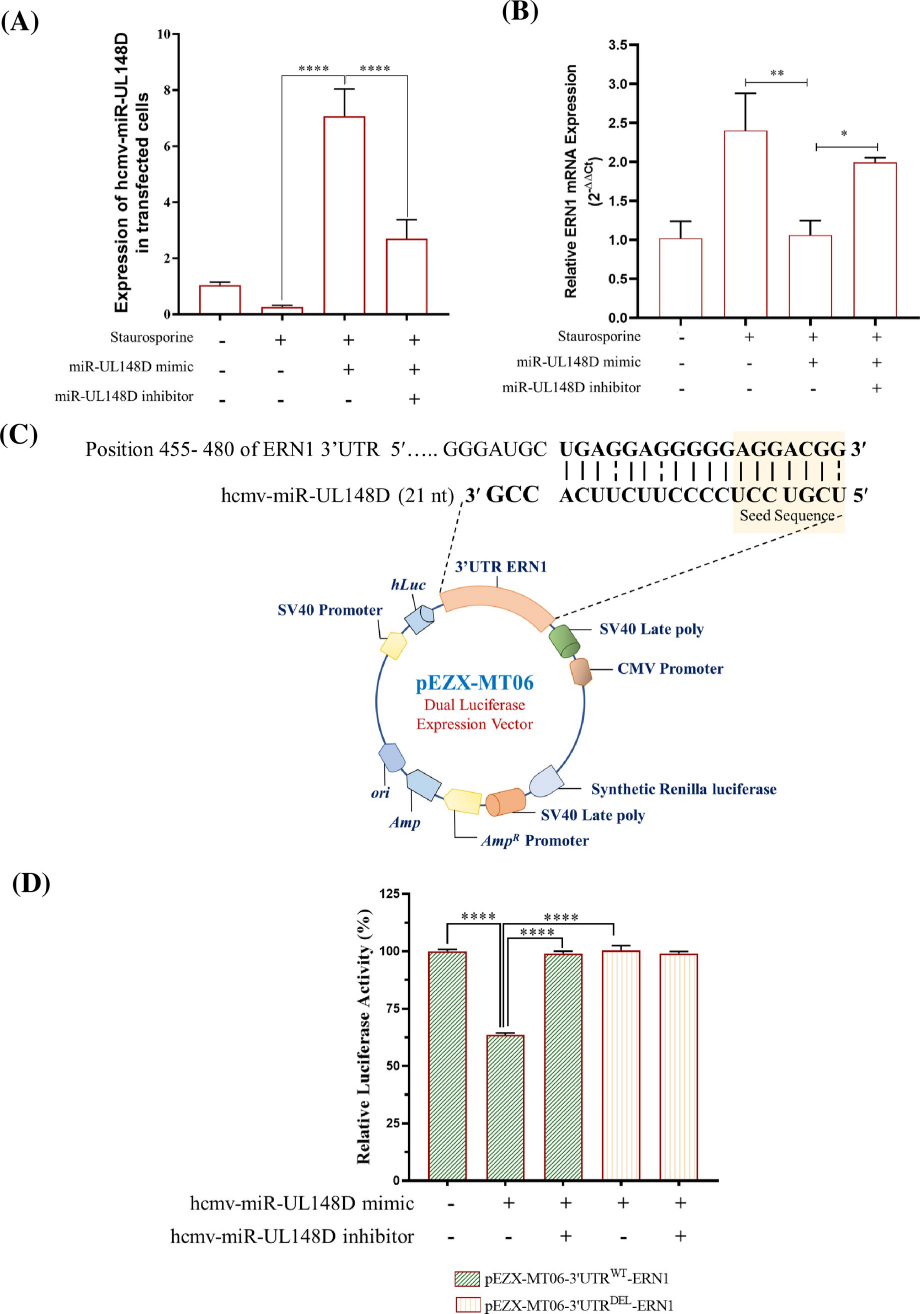
**(A)** Hcmv-miR-UL148D targets ERN1 through 3’UTR binding: **(A)** HEK293T cells were transfected with hcmv-miR-UL148D mimic or hcmv-miR-UL148D mimic and inhibitor together. RNA was isolated and the ectopic expression level of hcmv-miR-UL148D was measured through qRT-PCR, the miRNA levels were normalized to 5s rRNA in the corresponding samples. Result represents the mean ± SEM (n = 3 independent experiments) ****, p<0.0001. **(B)** hcmv-miR-UL148D downregulates the ERN1 mRNA expression. HEK293T cells were transfected with hcmv-miR-UL148D mimic or inhibitor followed by staurosporine treatment. RNA was isolated and expression of ERN1 was quantified and compared with respect to untreated cells. GAPDH was used as a housekeeping control and data plotted as a measure of relative expression. Results represent the mean ± SEM (n = 3 independent experiments) **p<0.01; ***p<0.001. **(C)** Cartoonistic representation of pEZX-MT06-3’UTR^WT/DEL^ dual-luciferase reporter vector, highlighting the binding site of hcmv-miR-UL148D. **(D)** hcmv-miR-UL148D targets the ERN1 3’UTR. HEK293T cells were transfected with hcmv-miR-UL148D mimic or inhibitor along with either pEZX-MT06-3’UTR^WT^-ERN1 or pEZX-MT06-3’UTR^DEL^-ERN1 luciferase reporter construct. Luciferase activity was measured 24 hr post transfection. Unlike the wild-type, the luciferase activity of the mutant pEZX-MT06-3’UTR^DEL^-ERN1 was not inhibited by hcmv-miR-UL148D.

Since microRNA functions through the regulation of its target, we asked whether the ERN1 is a functional target of hcmv-miR-UL148D. To test this hypothesis, we first confirmed the transfection efficiency and expression of hcmv-miR-UL148D through qRT-PCR by using specific primers in the presence of hcmv-miR-UL148D mimic or inhibitor in HEK293T cells treated with staurosporine. The level of hcmv-miR-UL148D significantly increases after transfection compared to the control group, while its expression was decreased when cells were transfected with hcmv-miR-UL148D inhibitor compared to the control, this result suggests that hcmv-miR-UL148D expressed efficiently in HEK293T cells and its inhibitor is efficiently functional (**Fig 3A**). Next, we measured the expression of ERN1 mRNA level in the presence of hcmv-miR-UL148D mimic or inhibitor in HEK293T cells treated with staurosporine. The RT-qPCR result suggests that staurosporine induced ERN1 mRNA level was significantly attenuated when HEK293T cells were transfected with hcmv-miR-UL148D mimic and attained the basal level. The specificity of regulation of ERN1 level through hcmv-miR-UL148D was further confirmed as augmented expression was observed in the presence of hcmv-miR-UL148D inhibitor (**Fig 3B**). These results suggest that hcmv-miR-UL148D directly regulates ERN1 at the mRNA level through degradation, suggesting that HCMV microRNA has an advantage over host microRNA, which is unlikely to degrade mRNA without pausing the translational machinery.

### 4. hcmv-miR-UL148D binds to the 3’UTR of ERN1

To address whether the hcmv-miR-UL148D downregulates the ERN1 levels through its 3′UTR and its degradation through RNAi is not clear. Our published *in silico* study indicates that the 3’UTR of ERN1 mRNA has a potential binding site at the position 455–480. In order to verify the binding and functionality of the hcmv-miR-UL148D site, we performed dual-luciferase reporter assays using the 3’UTR of ERN1 vector constructs. The wild type of ERN1 3′UTR designated as pEZX-MT06-3’UTR^WT^-ERN1 and the deleted binding site for hcmv-miR-UL148D designated as pEZX-MT06-3’UTR^DEL^-ERN1 were commercially procured from Genecopoeia, USA. The details of the vector constructs showing the binding site and mutated site are cartoonistically depicted in **Fig 3C**. The regulation of the ERN1 3′UTR was followed by transfecting HEK293T cells with the reporter constructs in the presence and absence of hcmv-miR-UL148D mimic or inhibitor. The luciferase activity of the ERN1 3′UTR was downregulated in the presence of hcmv-miR-UL148D mimic **(Fig 3D)**. The specificity of the regulation of ERN1 by hcmv-miR-UL148D was further confirmed by deleting the binding region for hcmv-miR-UL148D on 3′UTR of ERN1 i.e., 25nt long (from 455 to 480nt). Downregulation of luciferase activity was not observed when the binding site of hcmv-miR-UL148D was deleted in the 3′UTR of ERN1 (**Fig 3D**), supporting the view that ERN1 is a previously unrecognized target of hcmv-miR-UL148D. Collectively these results suggest that ERN1 is a bonafide target of hcmv-miR-UL148D.

### 5. hcmv-miR-UL148D downregulates the ERN1/IRE1α protein

**Fig 4:**
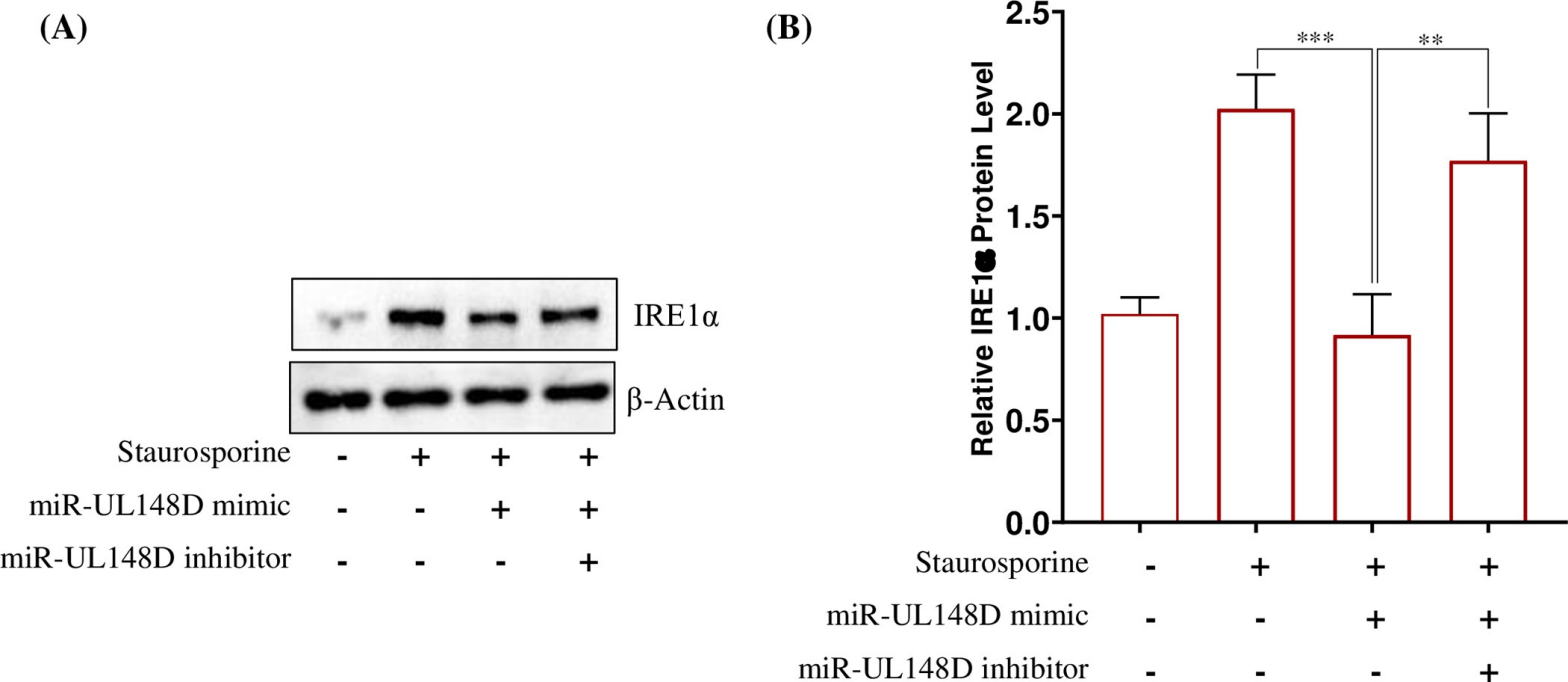
hcmv-miR-UL148D inhibits IRE1α protein level in staurosporine treated HEK293T cells. **(A)** The IRE1α protein levels were analyzed in different groups of cells transfected with hcmv-miR-UL148D, hcmv-miR-UL148D and its inhibitor followed by Staurosporine treatment. The IRE1α protein and the β-actin levels were examined through western blot. The bands of the blots have been cropped with no further manipulation. **(B)** Relative IRE1α protein levels in different groups of cells were quantified through ImageJ software. The IRE1α levels were normalized to β-actin and plotted as fold change with respect to control. Results represent the mean ± SEM (n = 3 independent experiments) **p<0.01; ***p<0.001.

Next, we asked whether binding of ERN1 mRNA by hcmv-miR-UL148D and its degradation results into the downregulation of ERN1 encoded protein IRE1α. We transfected HEK293T cells with either hcmv-miR-UL148D mimic or its inhibitor (25nM each) followed by staurosporine treatment. Then the total proteins were resolved and blotted against rabbit monoclonal anti-IRE1α and anti-β-actin antibodies as a loading control. The membrane was developed by the secondary anti-rabbit antibody conjugated with horseradish peroxidase. The cell group transfected with hcmv-miR-UL148D mimic reduces IRE1α protein level compared to the cell group treated with staurosporine only. Further, the reduction of IRE1α was abolished in the cell group simultaneously co-transfected with hcmv-miR-UL148D mimic and its inhibitor (**Fig 4A & 4B**). This result suggests that hcmv-miR-UL148D inhibits IRE1α protein level by degrading ERN1 mRNA.

### 6. hcmv-miR-UL148D-IRE1α-XBP1 axis regulates ER stress induced apoptosis in HEK293T cells

**Fig 5:**
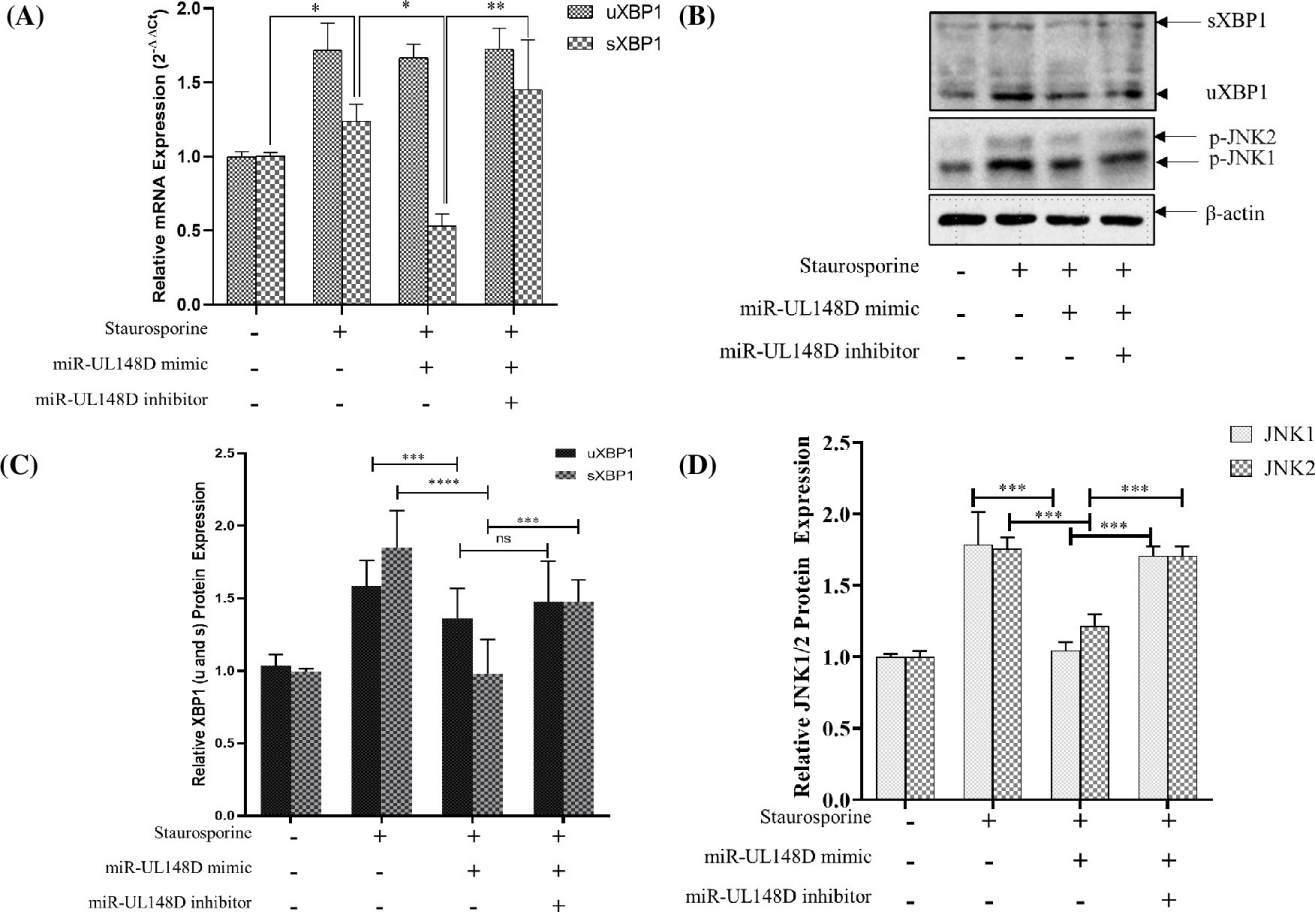
Hcmv-miR-UL148D regulates XBP1 splicing and JNK phosphorylation: (A) HEK293T cells were transfected with hcmv-miR-UL148D mimic or inhibitor followed by staurosporine treatment. RNA was isolated and XBP1 mRNA spliced (sXBP1) and unspliced (uXBP1) were measured through qRT-PCR. The uXBP1 and sXBP1 expression level were normalized to that of GAPDH and fold change was calculated and plotted with respect to the untreated control. Results represent the mean ± SEM (n = 3 independent experiments) *, p<0.05, **, p<0.01. **(B, C & D)** Whole cell lysate was prepared from the hcmv-miR-UL148D mimic or inhibitor transfection and staurosporine treated cells. The expression of p-JNK, uXBP1 and sXBP1 were analyzed in different groups of cells through western blot and β-actin was used as a loading control. The bands of the blots have been cropped with no further manipulation. The Relative protein expression levels in different groups of cells were analyzed through ImageJ software. Results represent the mean ± SEM (n = 3 independent experiments) **, p<0.01; ***, p< 0.001; ****P<0.0001; ns = non-significant.

Previous studies suggest that XBP1 has a role in ER stress [46, 47], however, little is known about the modulation and physiological significance of XBP1 in the context of hcmv-miR-UL148D. Once activated, mammalian IRE1α splices 26 nucleotides from the *Xbp1* mRNA, leading to a frameshift and the generation of XBP1s, that contains a C-terminal transactivation domain absent from the unspliced form XBP1u [48]. We studied the effect of hcmv-miR-UL148D on the splicing of XBP1 mRNA and strikingly we observed that hcmv-miR-UL148D mimic transfection downregulates the spliced XBP1 mRNA, however, there is no significant effect on the unspliced XBP1 mRNA (**Fig 5A**). Further, hcmv-miR-UL148D inhibitor increases the expression of spliced XBP1 mRNA (**Fig 5A**) which pinpoints the role of hcmv-miR-UL148D in XBP1 splicing. Since XBP1 mRNA does not have hcmv-miR-UL148D binding site suggesting that hcmv-miR-UL148D indirectly regulates XBP1 splicing. Since hcmv-miR-UL148D targets ERN1, which regulates XBP1 splicing [28], therefore we tested the hypothesis that hcmv-miR-UL148D regulates XBP1 splicing through ERN1. To strengthen our hypothesis, we transiently silence ERN1 in HEK293T cells for 24h followed by staurosporine treatment. We measured the splicing of XBP1 in the control and ERN1 silenced samples through qRT-PCR which suggests downregulation of sXBP1 level similar to hcmv-miR-UL148D mimic transfected samples (**Fig 5A**). This result suggests that ectopic expression of hcmv-miR-UL148D mimic translationally represses the IRE1α which results in the downregulation of XBP1 mRNA splicing and collectively inhibiting the ER-stress induced apoptosis. When the cells are under ER-stress they activate JNK pathway to initiate apoptosis and previous studies suggest that activated IRE1α phosphorylates JNK and thus activating the apoptosis effector molecule [49]. Therefore, we speculated that hcmv-miR-UL148D may regulate JNK1 activity as well. To test this, we transfected hcmv-miR-UL148D mimic or inhibitor and checked the phosphorylation status of JNK1 after staurosporine treatment. Interestingly, hcmv-miR-UL148D mimic suppresses phosphorylation level of JNK1 whereas hcmv-miR-UL148D inhibitor has the reverse effect (**Fig 5B**) suggesting that hcmv-miR-UL148D regulates apoptosis by controlling several downstream effector molecules and ERN1 is at the heart of this signaling pathway. These results clearly suggest that hcmv-miR-UL148D-IRE1α-XBP1 axis regulates staurosporine induced apoptosis in HEK293T cells (**Fig 5B–5D**).

### 7. Comparison in the downregulatory effect of siRNA of ERN1 and hcmv-miR-UL148D on the expression of ERN1 mRNA and its encoded protein

**Fig 6:**
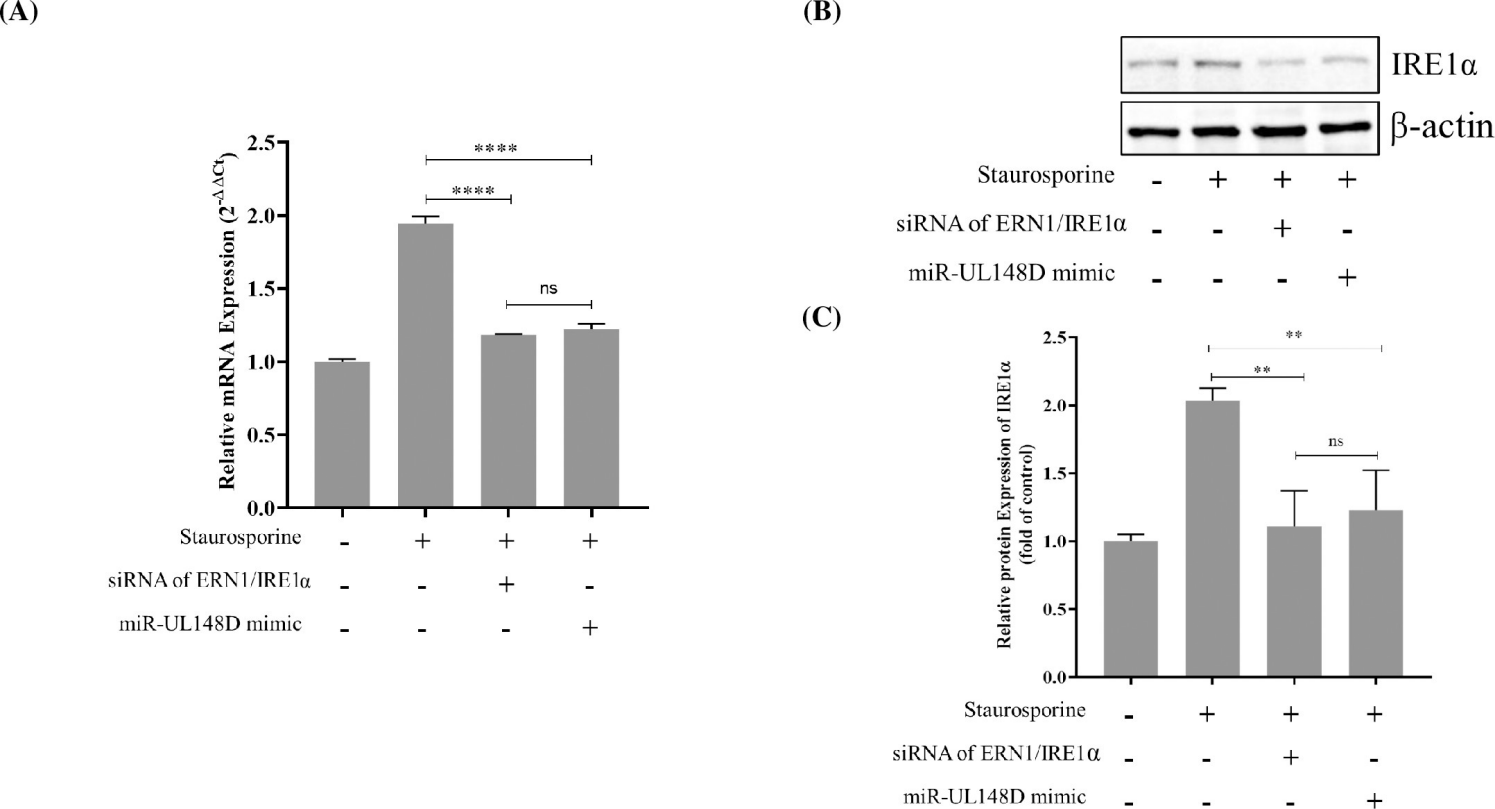
Comparison of downregulation of ERN1 by siRNA of ERN1 and hcmv-miR-UL148D: **(A) ERN1 mRNA downregulation:** The relative expression levels of ERN1 mRNA after the transfection of either hcmv-miR-UL148D and siRNA of ERN1 were measured through qRT-PCR. Results were expressed as the fold change (2^−ΔΔCt^) (± SEM; ****, p < 0.0001; ns = non-significant). (**B & C**) IRE1α protein encoded by ERN1 downregulation: The IRE1α protein downregulation after transfection with either siRNA of ERN1 and hcmv-miR-UL148D were analyzed through Western blot. The relative IRE1α protein quantification was performed through ImageJ software after normalizing with β-actin. Experiments were performed in triplicates (S2 Fig), and the data from three different experiments were used for statistical analysis (±SEM; **, p < 0.01, ns = non-significant).

The above studies confirm the downregulatory effect of hcmv-miR-UL148D mimic on ERN1 mRNA expression and encoded IRE1α protein. The comparison was done between hcmv-miR-UL148D mimic and ERN1 small interfering RNA (siRNA) designed against the 3’UTR of ERN1 (503–530 position of the 3′UTR of ERN1 as shown in S3 Fig). The HEK293T cells were transfected with either hcmv-miR-UL148D mimic (25nM) or ERN1 siRNA (25nM) followed by staurosporine treatment. The inhibition of ERN1 mRNA, and IRE1α protein downregulation were compared through qRT-PCR, and western blotting respectively. The resulting ERN1 mRNA **(Fig 6A)** and its encoded protein **(Fig 6B & 6C)** were plotted, which shows downregulatory effect of ERN1 siRNA as well as hcmv-miR-UL148D mimic on it. (±SEM; **, p < 0.01; ****, p<0.0001; ns = non-significant).

## Discussion

Given that miRNA regulates an overlapping set of targets and that a single target can be regulated by several miRNAs, there has been growing appreciation on the regulatory potential of miRNAs in diverse processes. Expectedly, there is now a body of research documenting the role of miRNAs in apoptosis and the interaction between host and pathogen. While contribution of host miRNA in the prevention of viral pathogenesis has been widely studied, it is poorly known how viral miRNA can confer viral survival inside the host. It is also established that human miRNA targets viral genes and functions as antiviral mediators to suppress viral pathogenesis [50, 51]. To evade/counter host defenses viruses might have further evolved microRNA mediated host gene silencing. These silencing can provide mechanisms to evade host defense and replication benefits [52]. Viruses selectively modulate cellular machinery for their effective replication and survival. The endoplasmic reticulum (ER) stress and unfolded protein response (UPR) pathways are regulated by the HCMV encoded proteins that benefit viral survival [26, 27, 53]. Viruses have evolved different strategies to allow viral genomic products that inhibit apoptosis to counteract antiviral immunity [54]. The present study demonstrates that the hcmv-miR-UL148D regulates the ER stress and UPR by targeting the ER stress signaling gene ERN1, which encodes IRE1α protein. Various HCMV miRNAs biological functions have been studied, including hcmv-miR-UL112-1 [55–57], hcmv-miR-US25-1 [56, 58], miR-US25-2-3p [58, 59] hcmv-miR-UL36-5p [14], hcmv-miR-UL70-3p and hcmv-miR-UL148D [8, 32], infers that the HCMV utilizes its miRNAs to regulate its own genes as well as the host cell genes during infection, to achieve immune evasion, regulation of cellular processes, viral DNA replication and counteracting of cellular apoptosis.

Staurosporine is a well-known apoptotic agent [44] which also induces ER-stress and ER-stress apoptosis [59]. The staurosporine treatment in HEK293T cells increased the mRNA expressions of apoptotic genes such as ERN1, MOAP1, BAK1, Caspase 3 and Caspase 7 (**Fig 1**). The UPR plays a significant role in determining the cell fate to survive or death [60], here we find that UPR is involved in staurosporine treatment in HEK293T cells by triggering the expression of IRE1α/XBP1/c-JNK which is downregulated by the hcmv-miR-UL148D (**Figs 4 & 5**). In humans IRE1 found in two isoforms, IRE1α and IRE1β, highly conserved ER stress sensor, involved in deciding the cell fate to survive or death due to apoptosis [61, 62]. It controls the cell survival/apoptosis based on the severity of ER stress [62]; under unrelieved ER-stress, it induces apoptosis [28, 63]. Viruses exhibits multiple mechanisms to regulate IRE1α signaling, thereby facilitate their replication and survival in the cell. Some viruses like Hepatitis B virus, Influenza A virus, Japanese encephalitis virus, and Flavivirus activate IRE1-XBP1 signaling [62, 64–66], while Hepatitis C virus and Rotavirus suppress this pathway [67, 68]. In this study, we demonstrated that HCMV miR-UL148D inhibits IRE1α-XBP1 and IRE1α-JNK signaling, leading to suppression of apoptosis **(Fig 2A–2D),** and this anti-apoptotic effect of miR-UL148D was confirmed by using its inhibitor. The ectopic expression of hcmv-miR-UL148D degrades ERN1 mRNA through binding its 3’ UTR which suggests that ER-stress apoptosis induced by staurosporine, was inhibited by the translational repression of the ERN1 (**Fig 3A, 3B and 3D**). The HCMV reported to regulate the UPR and thereby regulate the ER stress induced apoptosis through its proteins. The HCMV anti-apoptotic protein, pUL38, suppress the ER-stress induced cell death by inhibiting the phosphorylation of c-Jun N-terminal kinase (JNK), which was mediated through IRE1 [27]. The HCMV proteins modulate the UPR, for example the anti-apoptotic HCMV proteins, pUL37x1 reported to induces the UPR [26], and pUL148 activates the UPR [69], suggesting the HCMV proteins modulate the UPR in accordance to its survival or replication benefit.

The staurosporine induced XBP1 splicing was significantly downregulated by the hcmv-miR-UL148D mimic treatment (**Fig 5**) suggests that the hcmv-miR-UL148D actually reduced the IRE1α protein, which leads to the downregulation of XBP1 splicing, as IRE1α participates in unconventional XBP1 splicing. The XBP1s translates to an active XBP1 protein, which is a transcription factor, and initiate the transcription of proteins involved in ER-associated protein degradation (ERAD) upon binding to the DNA. The IRE1α/ XBP1 pathway is dominant to promote apoptosis [65, 70–72]. When the UPR fails to resolve the ER- stress, the IRE1 pathway shifts to activate the stress kinases, Jun -n- terminal kinase (JNK) and p38 MAPK resulting into ER-stress induced apoptosis [28]. Activation of JNK by UPR not only contributes to apoptosis, but through regulation of cytokines may attract the phagocyte to engulf the infected-apoptotic cells. Overall, hcmv-miR-UL148D inhibition of apoptosis suggests that it helps the HCMV for its effective replication and spreading in the host cells. Alternatively, ER quality control proteins regulated by the IRE1α-XBP1 pathway promotes virus replication by enhancing the viral proteins modification, folding, and trafficking. Another possibility is that XBP1s stimulates the phospholipid biosynthesis and ER expansion [73], thus providing the lipid that is necessary for the enveloped virus particle assembly.

Regulation of UPR and ER stress induced apoptosis through silencing the IRE1α mRNA was reported in *vivo* and *in vitro* [63]. The human miRNA, hsa-miR-34a-5p inhibits the tunicamycin induced UPR by targeting and inhibiting the IRE1α protein [74]. The present study is also in accordance with the above studies that ER-stress induced apoptosis downregulation due to the inhibition of the IRE1α protein. Further, many HCMV miRNAs demonstrated the anti-apoptotic effects *in in vitro* [8, 12, 14–17, 31, 32], however, none of them shown to function through ER stress-induced apoptosis. Wang et al reported the antiapoptotic activity of hcmv-miR-UL148D *in vitro* but function through IEX1 [12], but in our studies the functional target found to be the IRE1α. This result suggests that the hcmv-miR-UL148D function is context specific and downregulates apoptosis stimulated through various agents by targeting the different apoptotic genes. Further, this study sheds light on the coordination between the HCMV miRNAs and its proteins. The HCMV protein pUL38 inhibits the phosphorylation of JNK which is essential for ER-stress induced apoptosis [27], and the hcmv-miR-UL148D downregulates the IRE1α which is required for ER stress induced apoptosis.

In all, this study provides comprehensive insights into the mechanisms of UPR induced apoptosis and a new regulatory layer by hcmv-miR-UL148D which ultimately provides HCMV replication advantage inside the cell. Further studies required to elucidate how exactly and to what extent the hcmv-miR-UL148D miRNAs regulate the ER stress-induced cellular apoptosis.

## Conclusions

Growing evidence indicates that HCMV uses its miRNA machinery in modulating the apoptosis and they exert their function in a concordance or synergistic with HCMV proteins. The present study sheds light on the HCMV miRNAs role on ER-stress induced apoptosis, as it demonstrates the negative regulation of ER-stress-induced apoptosis by the hcmv-miR-UL148D, by targeting and downregulating the ER- stress sensing signaling molecule, i.e., ERN1 mRNA and its encoded protein IRE1alpha. To the best of our knowledge, it is the first report on HCMV miRNA regulatory role on ER-stress induced apoptosis.

## Supporting information

S1 Fig Triplicates of the western blots for ERN1/IRE1α downregulation by the hcmv-miR-UL148D. (TIF)

S2 Fig Triplicates of the western blots for ERN1/IRE1α downregulates by the siRNA of ERN1/IRE1α and hcmv-miR-UL148D. (TIF)

S3 Fig The 3’UTR of ERN1 shows the binding sites for the siRNA of ERN1/IRE1α and hcmv-miR-UL148D. (TIF)

S4 Fig Duplicates of the western blots for XBP1u/s, p-JNK1/2 downregulation by the hcmv-miR-UL148D. (TIF)

S1 Raw images (PDF)
