# Supplementary figures and images for: Hcmv-miR-UL148D regulates the staurosporine-induced apoptosis by targeting the Endoplasmic Reticulum to Nucleus signaling 1(ERN1)
Source: PLoS One. 2022 Sep 26;17(9):e0275072. doi: 10.1371/journal.pone.0275072 (PMC9512192; doi:10.1371/journal.pone.0275072)

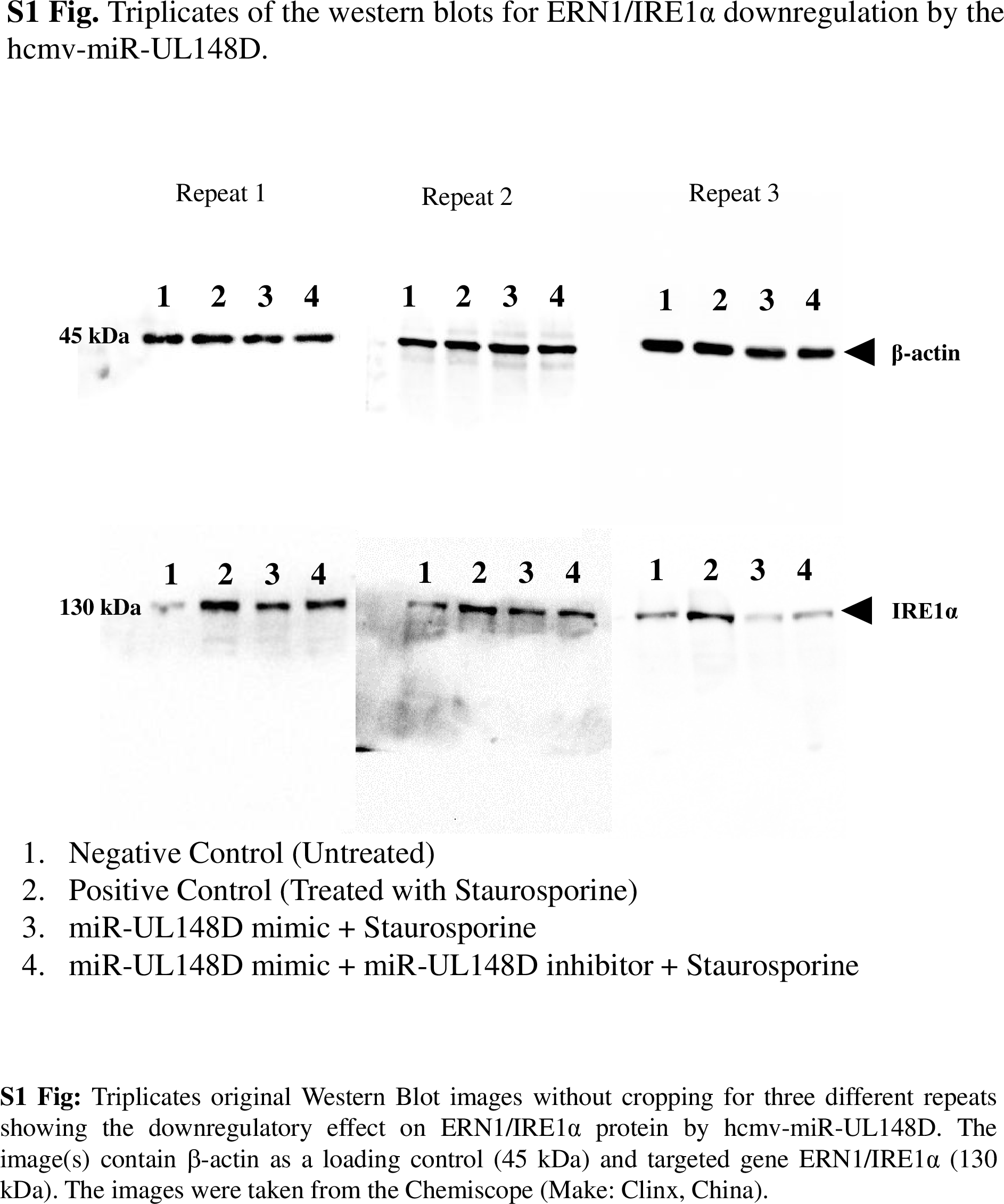

Supplement: S1 Fig — (TIF) [file pone.0275072.s001.tif]

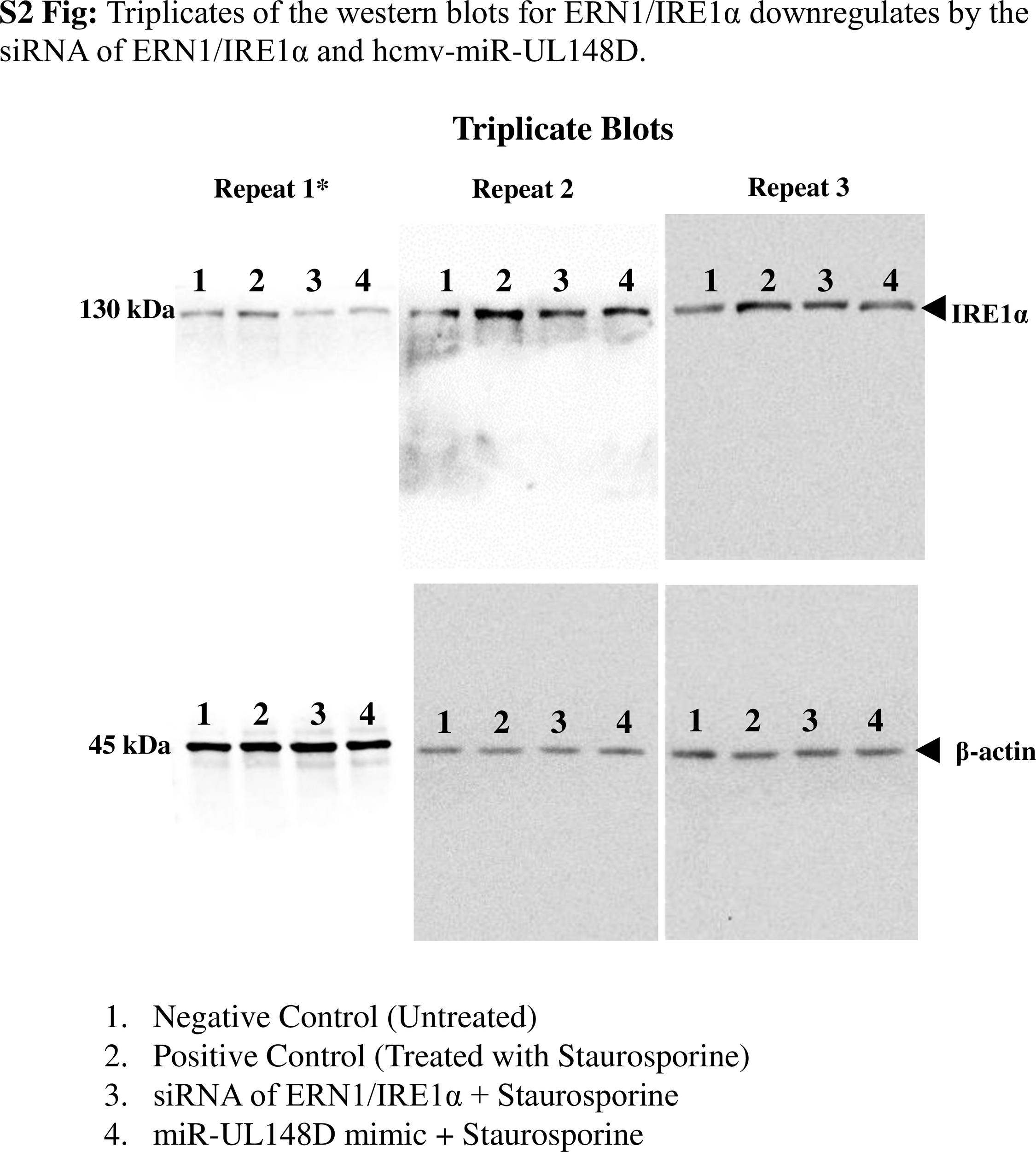

Supplement: S2 Fig — (TIF) [file pone.0275072.s002.tif]

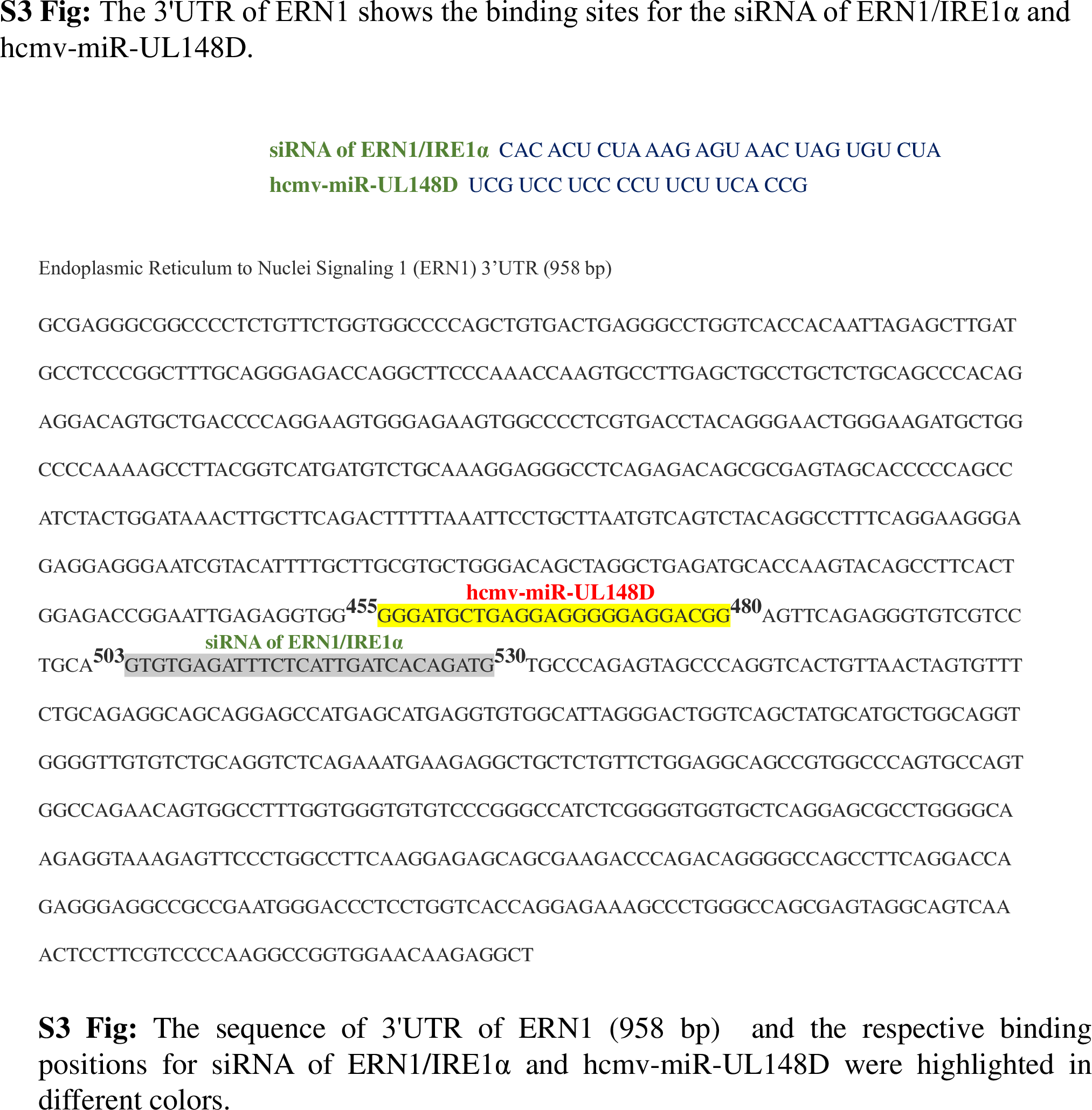

Supplement: S3 Fig — (TIF) [file pone.0275072.s003.tif]

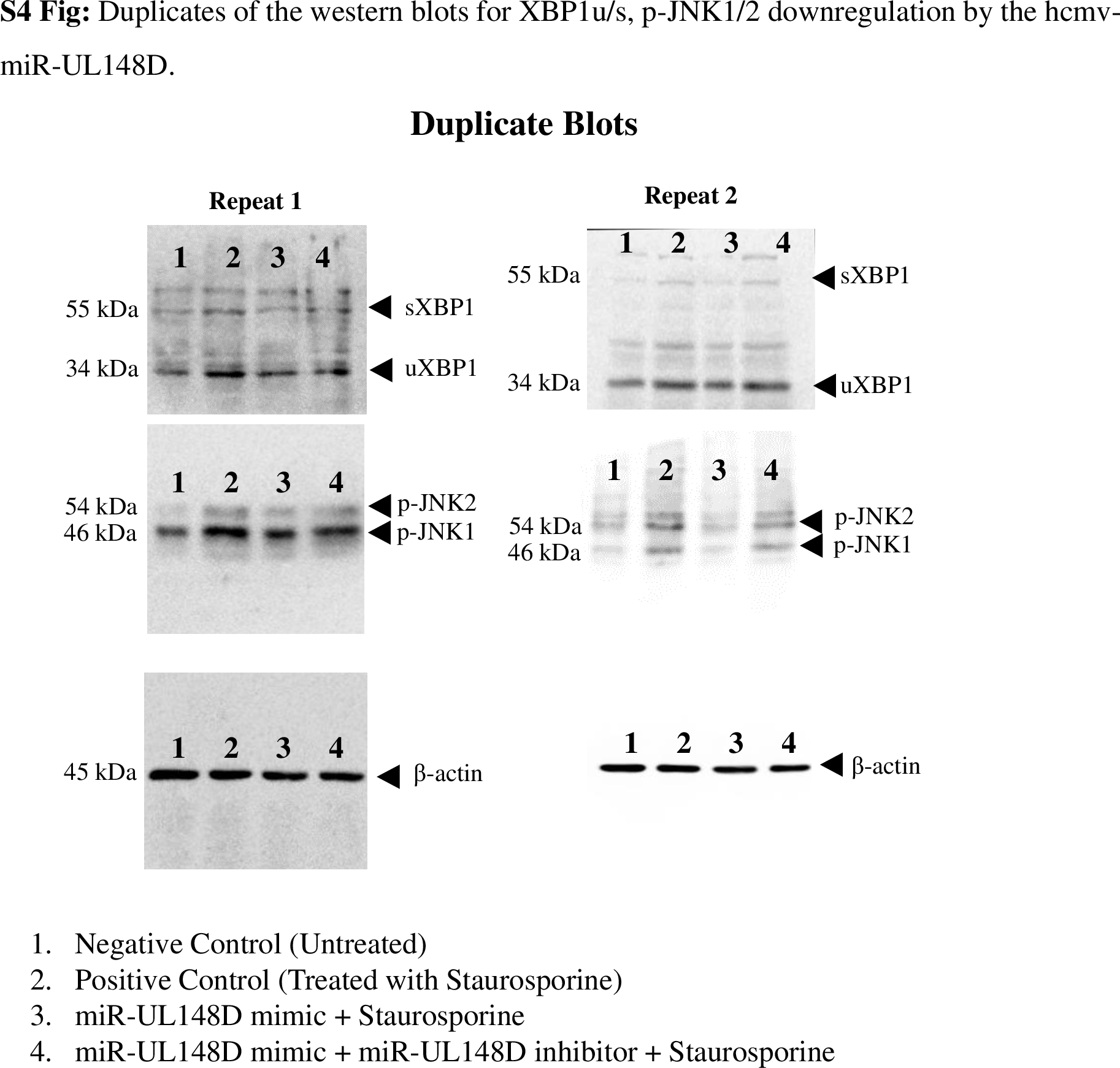

Supplement: S4 Fig — (TIF) [file pone.0275072.s004.tif]
