# Supplementary material for: Hcmv-miR-UL148D regulates the staurosporine-induced apoptosis by targeting the Endoplasmic Reticulum to Nucleus signaling 1(ERN1)
Source: PLoS One. 2022 Sep 26;17(9):e0275072. doi: 10.1371/journal.pone.0275072 (PMC9512192; doi:10.1371/journal.pone.0275072)
Supplement: S1 Raw images — (PDF) [file pone.0275072.s005.pdf]

Raw Fig. 1

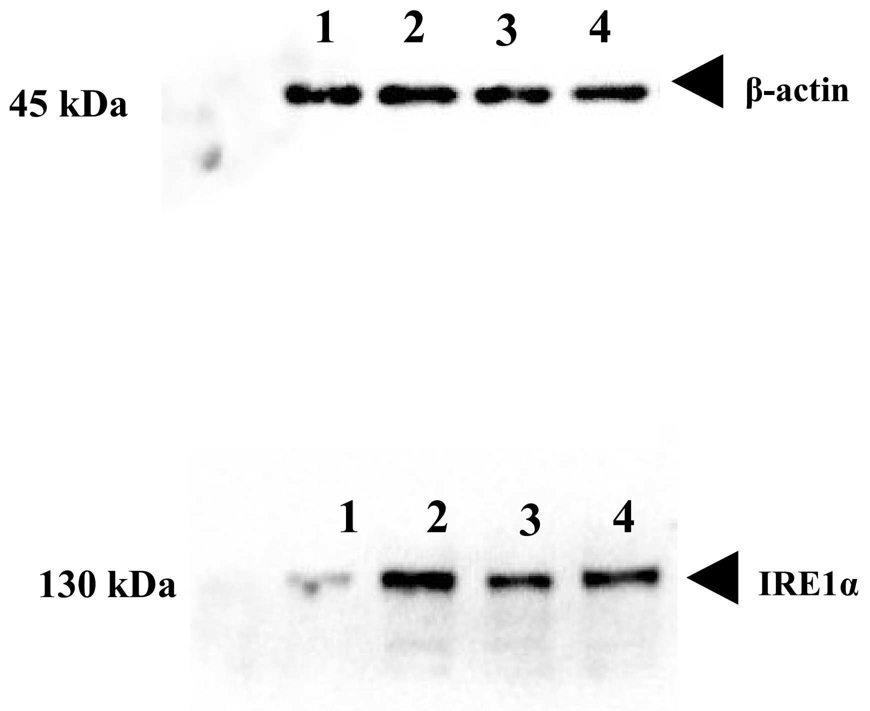

Where

1. Negative Control (Untreated)
2. Positive Control (Treated with Staurosporine)
3. miR-UL148D mimic + Staurosporine
4. miR-UL148D mimic + miR-UL148D inhibitor + Staurosporine

Incorporated as Fig 4 A

Raw Fig. 2

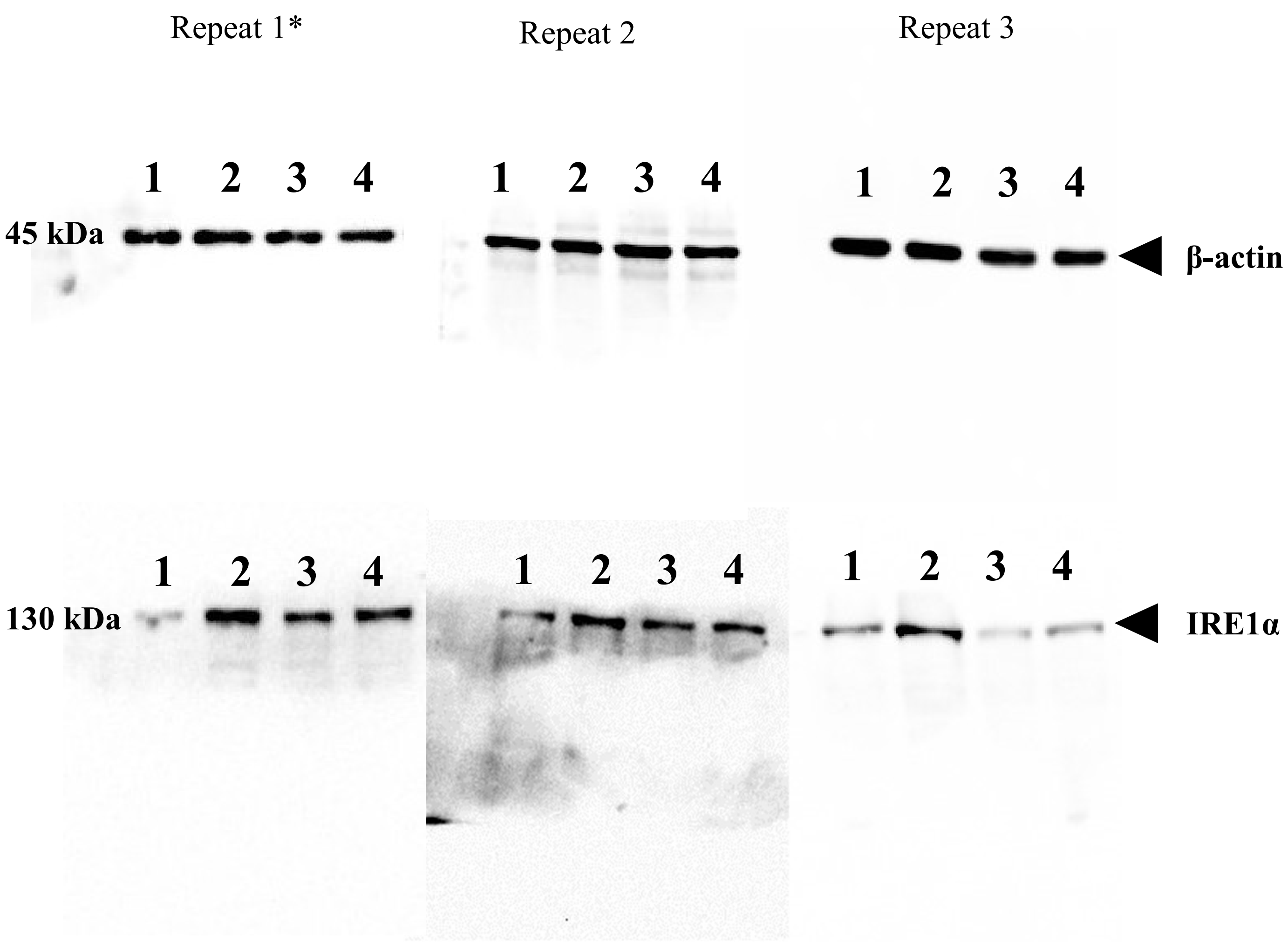

Where

1. Negative Control (Untreated)
2. Positive Control (Treated with Staurosporine)
3. miR-UL148D mimic + Staurosporine
4. miR-UL148D mimic + miR-UL148D inhibitor + Staurosporine

***Incorporated as Fig 4 A**

Raw Fig. 3

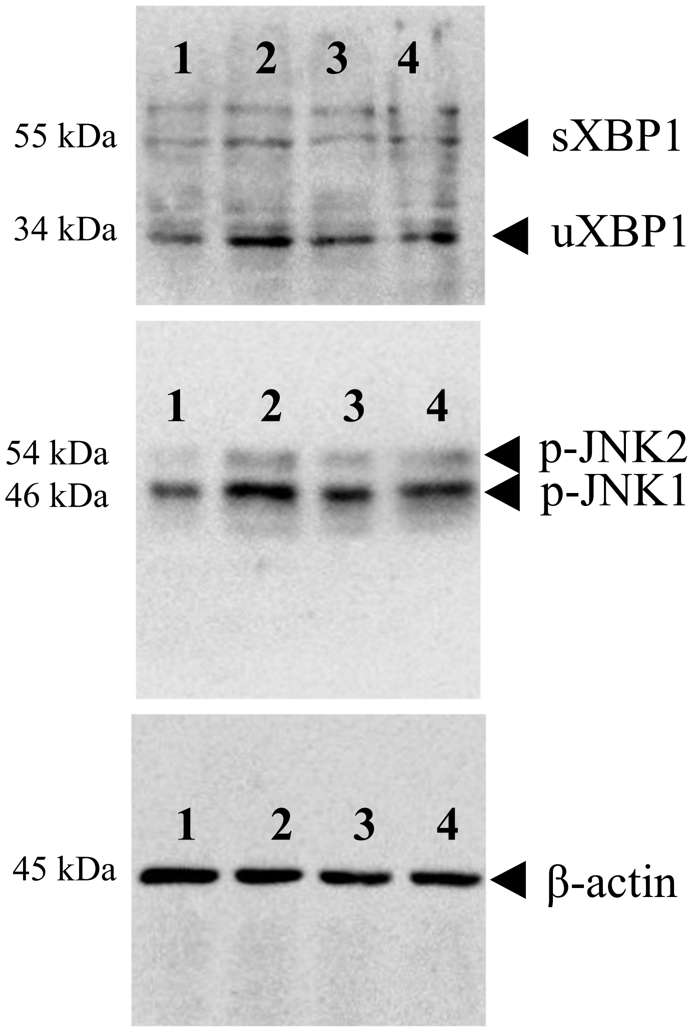

1. Negative Control (Untreated)
2. Positive Control (Treated with Staurosporine)
3. miR-UL148D mimic + Staurosporine
4. miR-UL148D mimic + miR-UL148D inhibitor + Staurosporine

Incorporated as Fig 5 B

Raw Fig. 4

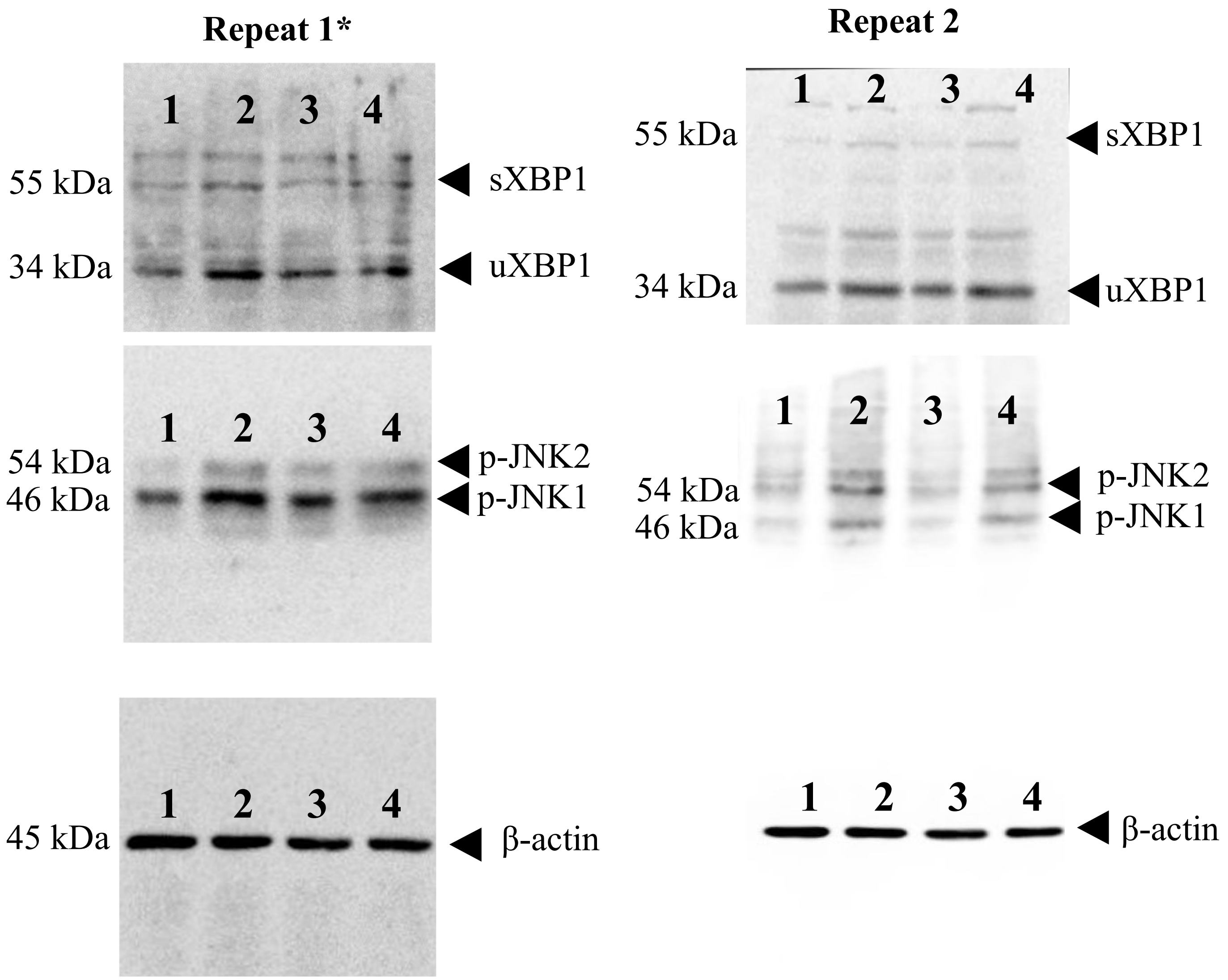

1. Negative Control (Untreated)
2. Positive Control (Treated with Staurosporine)
3. miR-UL148D mimic + Staurosporine
4. miR-UL148D mimic + miR-UL148D inhibitor + Staurosporine

***Incorporated as Fig 5 B**

Raw Fig. 5

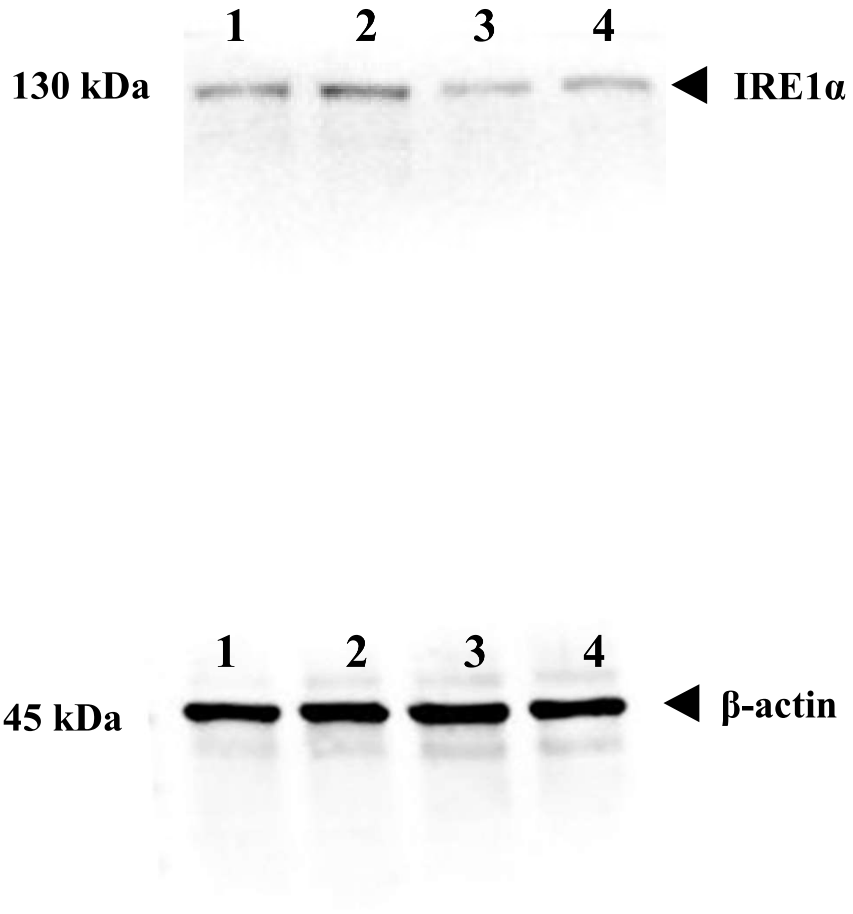

1. Negative Control (Untreated)
2. Positive Control (Treated with Staurosporine)
3. siRNA of ERN1/IRE1α + Staurosporine
4. miR-UL148D mimic + Staurosporine

Incorporated as Fig 6 B

Raw Fig. 6

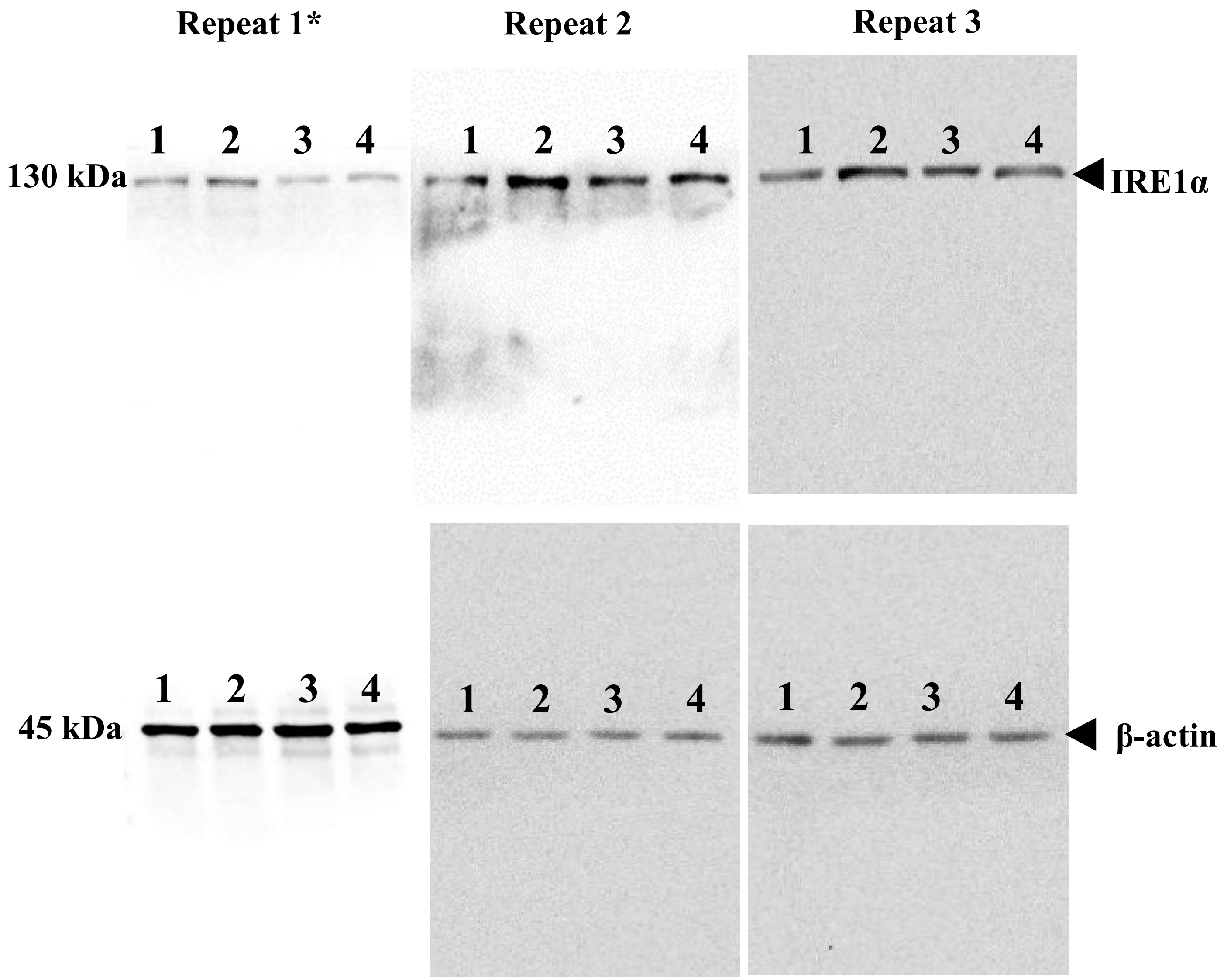

1. Negative Control (Untreated)
2. Positive Control (Treated with Staurosporine)
3. siRNA of ERN1/IRE1α + Staurosporine
4. miR-UL148D mimic + Staurosporine

***Incorporated as Fig 6 B**
